# Tracking Permeation of Dimethyl Sulfoxide (DMSO) in *Mentha × piperita* Shoot Tips Using Coherent Raman Microscopy

**DOI:** 10.3390/plants12122247

**Published:** 2023-06-08

**Authors:** Heidi D. Kreckel, Fionna M. D. Samuels, Remi Bonnart, Gayle M. Volk, Dominik G. Stich, Nancy E. Levinger

**Affiliations:** 1 Department of Chemistry, Colorado State University, Fort Collins, CO 80523, USA; fionna.samuels@colostate.edu; 2 USDA ARS National Laboratory for Genetic Resources Preservation, Fort Collins, CO 80521, USA; remi.bonnart@usda.gov (R.B.); gayle.volk@usda.gov (G.M.V.); 3 Advanced Light Microscopy Center, University of Colorado, Denver, CO 80045, USA; dominik.stich@cuanschutz.edu; 4 Department of Electrical and Computer Engineering, Colorado State University, Fort Collins, CO 80523, USA

**Keywords:** plant cryopreservation, cryoprotectant distribution, peppermint, Raman, CARS microscopy, brightfield microscopy

## Abstract

Cryopreservation has emerged as a low-maintenance, cost-effective solution for the long-term preservation of vegetatively propagated crops. Shoot tip cryopreservation often makes use of vitrification methods that employ highly concentrated mixtures of cryoprotecting agents; however, little is understood as to how these cryoprotecting agents protect cells and tissues from freezing. In this study, we use coherent anti-Stokes Raman scattering microscopy to directly visualize where dimethyl sulfoxide (DMSO) localizes within *Mentha × piperita* shoot tips. We find that DMSO fully penetrates the shoot tip tissue within 10 min of exposure. Variations in signal intensities across images suggest that DMSO may interact with cellular components, leading to its accumulation in specific regions.

## 1. Introduction

Plant genebanks maintain collections of crop genetic diversity that are critical for global food security. These invaluable collections must be backed up to prevent losses that can result from biotic and abiotic threats. Seed collections can often be placed in cold storage at secondary locations; however, many vegetatively propagated fruit, nut, and vegetable collections are maintained in field repositories, greenhouses, or tissue culture as the desired cultivars cannot be preserved through seed banking [1]. These actively growing collections are expensive to maintain at multiple locations and are at risk of contamination, plant pathogens, and pests [2,3]. Cryopreservation of shoot tips and dormant buds provides a safe, secure, and cost-effective method to back up vegetatively propagated genebank collections [1,4,5].

During cryopreservation, living, regenerable tissues are stored at or near liquid nitrogen temperatures (−196 °C), from which they can then be warmed and grown into plants identical to the original source materials after years of storage [4,6]. One-millimeter shoot tips can be cryopreserved using droplet vitrification techniques that enlist mixtures of highly concentrated cryoprotecting agents (CPAs) to dehydrate and increase the viscosity within cells [7]. CPAs are essential to forming a vitrified state, an amorphous glassy state, during rapid cooling of shoot tips, which protects them from injuries that result from the formation of ice crystals within cells [8]. The most commonly used mixtures in plant cryopreservation are plant vitrification solution 2 (PVS2), consisting of 15% (*w*/*v*) dimethyl sulfoxide (DMSO), 15% (*w*/*v*) ethylene glycol, 30% (*w*/*v*) glycerol, and 0.4 M sucrose, and plant vitrification solution 3 (PVS3), consisting of 50% (*w*/*v*) glycerol and 50% (*w*/*v*) sucrose [8,9,10]. Despite the demonstrated success of these CPAs, they are not universally cryoprotective. For example, peppermint—which has a well-established droplet vitrification cryopreservation protocol [11]—only has an average survival rate of 73% [6,12,13]. The research presented here aims to reveal the fundamental properties of CPAs by determining their localization within living plant shoot tips.

Past studies have investigated both the response of shoot tips to CPA exposure and fundamental interactions between cells and CPAs [14,15,16,17,18,19,20,21,22,23,24,25,26,27,28]. Light microscopy studies have observed plasmolysis and deplasmolysis within cells and tissues exposed to CPAs [14,15,16]. The time for plasmolysis and deplasmolysis to complete within these cells and tissues is often used as a metric for the time it takes for CPAs to fully enter cells and tissues; however, recent work by our group has shown that CPAs permeate the cell membrane well before deplasmolysis begins [17]. This suggests that CPA permeation alone is not responsible for deplasmolysis and that permeation occurs more quickly than brightfield studies of deplasmolysis suggest. Other studies have focused on the viability of shoot tips following exposure to various mixtures of CPAs to determine what works best for each cultivar [15,18,19]. Although this process has been effective, it relies on an empirical methodology that is time-consuming and laborious. While several reports of mint cryopreservation protocols have appeared for more than 30 years [12,29,30,31,32], researchers continue to use empirical methods to find highly effective protocols [6,12,29,30,32]. Other studies have explored the effectiveness of DMSO as a component of CPA mixtures, such as PVS2 [6,12,29,30,32]. Additional studies have explored the fundamental thermal properties of CPAs [20] and their potential interactions with cell membranes [21,22,23,24,25] and proteins [26,27,28] providing some quantitative information to the cryo-community. However, all these studies lack direct evidence of CPA permeation as they interact with living cells and tissues. 

Fluorescence microscopy is a popular method for tracking molecules as they travel through living systems. Although fluorescently tagged molecules can be easily visualized, the bulky fluorophores required may prevent accurate tracking of smaller CPAs, such as DMSO, ethylene glycol, and glycerol. Further, fluorescent techniques often use visible light that can result in photobleaching, cell damage, and death when absorbed by living tissues over long periods of time [33]. Coherent anti-Stokes Raman scattering (CARS) microscopy is a non-invasive technique that does not rely on bulky fluorophores or molecular tags that may perturb a sample. Rather, it produces an image from the signal of a single, unique vibrational mode of a molecule [33]. Further, CARS microscopy often uses near- to mid-IR light that allows for greater penetration into thicker samples and reduced cell damage over long-term exposure [33]. These features make CARS microscopy an effective technique for our study.

Previously, we demonstrated CARS microscopy as an effective method to measure the perfusion of DMSO into rice callus cells [34]. Our work shows that when exposed to aqueous DMSO solution, rather than perfusing uniformly in the cells, the DMSO sequesters into pockets within the cells, hypothesized to be starch granules. Those findings cannot necessarily be extrapolated to organized tissue systems, such as shoot tips, but they provide evidence of CPA permeation into live plant cells. 

Here we report the application of CARS microscopy to measure the localization and distribution of deuterated DMSO (d_6_-DMSO) within living peppermint (*Mentha × piperita*) shoot tips. We compare CARS microscopy results with brightfield microscopy to show how the overall response of the shoot tips to DMSO, an example CPA. Visualization of the d_6_-DMSO indicates that the CPA component is not evenly distributed within the shoot tips, further indicating that DMSO may also be unevenly distributed within cells. 

## 2. Results

### 2.1. Brightfield Imaging

**Figure 1:**
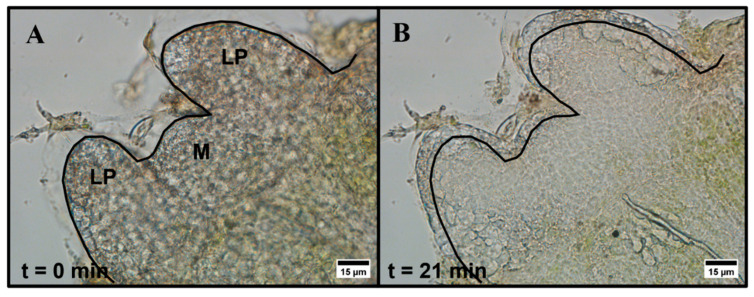
Brightfield microscopy images of peppermint shoot tip (**A**) prior to exposure and (**B**) 21 min after exposure to 15% (*w*/*v*) DMSO in aqueous media. LP = leaf primordia region, M = meristem region. The black line indicates the initial edge of the shoot tip prior to exposure. Images were collected using a 40×, 0.60 NA air-immersion objective. Image size: 1600 × 1200 pixels. Pixel size: 0.11 μm/pixel.

Entire one-millimeter peppermint shoot tips were exposed to aqueous 15% (*w*/*v*) DMSO in a perfusion chamber and observed using brightfield microscopy. Within the first 5 min of exposure to 15% (*w*/*v*) DMSO, shoot tips either contract and re-expand or only expand slightly. We also observe subcellular motion, but due to the nature of the tissue, we cannot resolve the exact response of individual cells. All six individual shoot tips we measured swell and remain expanded in the 20–25 min observation timeframe with no further changes, but the extent of the expansion varies among shoot tip samples (Figure 1 and Figure S1). The expansion often appears primarily in the leaf primordia regions and, to a lesser extent, in the meristematic region. 

### 2.2. Vibrational Spectroscopy

**Figure 2:**
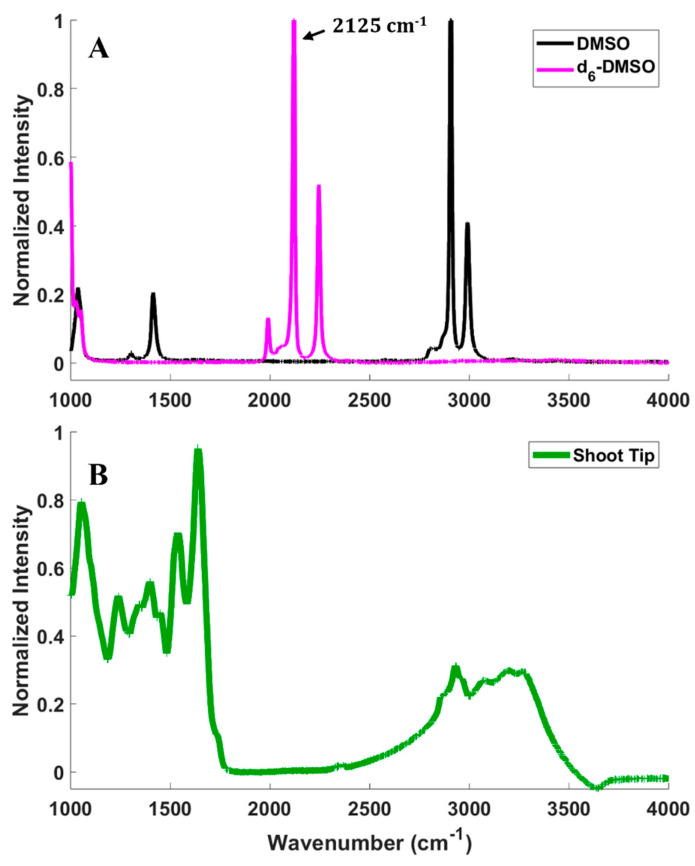
(**A**) Spontaneous Raman spectra of pure d_6−_DMSO (magenta) and DMSO (black). (**B**) IR absorption spectrum of a peppermint shoot tip (green).

CARS microscopy imaging relies on the detection of a signal from a molecular vibrational mode of the d_6_-DMSO introduced to samples, so it is essential to confirm the vibrational spectroscopy of DMSO, deuterated DMSO (d_6_-DMSO), and of a peppermint shoot tip (Figure 2). Figure 2A presents the Raman spectra of pure DMSO and d_6_-DMSO. The spectral region between approximately 1900 and 2600 cm^−1^ is devoid of fundamental molecular vibrational modes for most common molecules [35], but substituting hydrogen with deuterium on the DMSO replaces the CH_3_ groups with CD_3_ and shifts the vibrational frequencies into this silent region of the spectrum (see Figure 2A). In our previous work, we measured the Raman spectrum of rice callus cells compared to DMSO and d_6_-DMSO and showed that the rice cells contribute little intensity in the 1900–2600 cm^−1^ region [34]. However, fluorescence from the peppermint shoot tip prevented the collection of its Raman spectrum. To confirm that the d_6_-DMSO C-D vibrational mode exists in a vibrationally quiet region of the shoot tip spectrum, we collected an infrared (IR) spectrum of a peppermint shoot tip (Figure 2B). Although Raman and IR spectral features can have differing intensities, the traditionally quiet region coupled with the shoot tip IR spectrum gives us confidence that the d_6_-DMSO vibrations appear in a region where the shoot tip displays no vibrational bands, thus confirming that CARS signals collected in that region arise exclusively from the d_6_-DMSO. To maximize the CARS intensity in imaging experiments, we imaged at 2125 cm^−1^, the frequency corresponding to the highest intensity in the d_6_-DMSO spectrum (Figure 2A).

### 2.3. CARS Imaging

**Figure 3:**
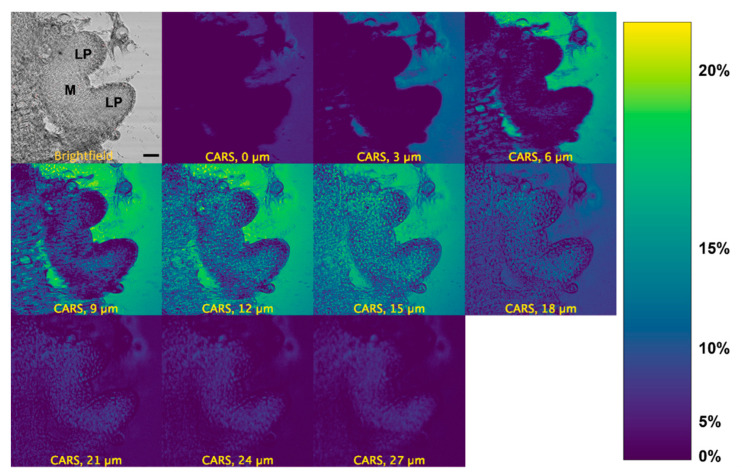
CARS montage for a z-stack of a peppermint shoot tip after >10 min exposure to 15% (*w*/*v*) d_6_-DMSO. Top left: brightfield image of the shoot tip identifying leaf primordia (LP) and meristem (M) regions. All others: CARS images acquired from the same shoot tip at depths of 0, 3, 6, 9, 12, 15, 18, 21, 24, and 27 µm. The second panel from the left in the top row represents an image taken from the top of the shoot tip; subsequent images are presented, in order, from the top to the bottom of the shoot tip. Right: Calibration bar generated by a calibration curve correlating mean pixel intensity to aqueous d_6_-DMSO concentration (see Figure S2). Images were collected using a 20×, 0.75 NA air immersion objective. Vibrational frequency: 2125 cm^−1^. Image size: 800 × 800 pixels. Pixel size: 0.12 μm/pixel Scale bar = 10 μm.

We measured the localization of d_6_-DMSO in the peppermint shoot tips using its C-D vibrational signature. We observe a d_6_-DMSO signal across multiple layers of the shoot tip approximately 10 min after exposure to an aqueous 15% (*w*/*v*) d_6_-DMSO solution (Figure 3). A brightfield image of the same shoot tip identifies the major regions of interest, e.g., leaf primordia and meristem (Figure 3, top left). CARS images for additional shoot tip samples appear in Figures S3 and S4. The calibration bar (Figure 3, right) correlates color in the images to approximate d_6_-DMSO concentrations. Concentrations were estimated by correlating mean pixel intensity in images acquired from calibration samples of 0, 5, 10, 15, and 20% (*w*/*v*) aqueous d_6_-DMSO solutions and fitting the resulting points to a second-order polynomial (Figure S2). 

The strongest CARS signal from d_6_-DMSO appears in the central depths of each shoot tip (see Figure 3, Figures S3 and S4). Images collected near the top and bottom of the shoot tip (see Figure S9 for definition) show little CARS signal while higher CARS signals appear in the shoot tip interior; the highest signals in Figure 3 appear at 12 and 15 µm. Low CARS intensity near the top and bottom of the sample reflects technical aspects of the image acquisition. That is, the depth from which the CARS signal comes is much broader than the spacing between individual depths collected in the z-stack. Near the bottom and top of the shoot tip, the laser focus distributes only partially in the region, including plant tissue and surrounding media; laser light interacting with the glass slide or air generates no CARS signal, thus lowering the overall signal collected. Additional technical aspects may contribute to the loss of the CARS signal; these aspects are discussed further in the Supplementary Materials. Thus, we concentrate image interpretation on the central depths of the shoot tips. 

**Figure 4:**
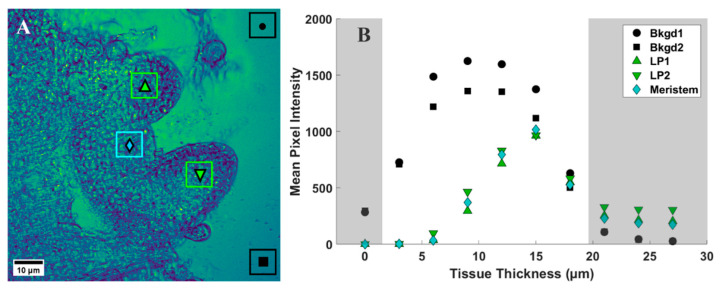
(**A**) Representative CARS z-stack image (depth of 15 µm, Figure 3) of a peppermint shoot tip following exposure to 15% (*w*/*v*) d_6_-DMSO. Colored boxes, each containing a unique symbol, represent selected regions of interest (ROIs). Black ROIs = background regions, or regions without plant tissue; green ROIs = leaf primordia; cyan ROIs = meristem. The unique symbols in each ROI correspond to data points shown in B and Figure 5. Each ROI contains 5400 pixels. (**B**) Mean pixel intensity recorded for each ROI indicated in A for each tissue thickness (corresponding images in Figure 3). Shaded regions represent areas of low signal intensity due to technical aspects of CARS imaging (see Supplementary Materials) and are not considered in our analyses. Images were collected using a 20×, 0.75 NA air immersion objective. Vibrational frequency: 2125 cm^−1^. Image size: 800 × 800 pixels. Pixel size: 0.12 μm/pixel.

**Figure 5:**
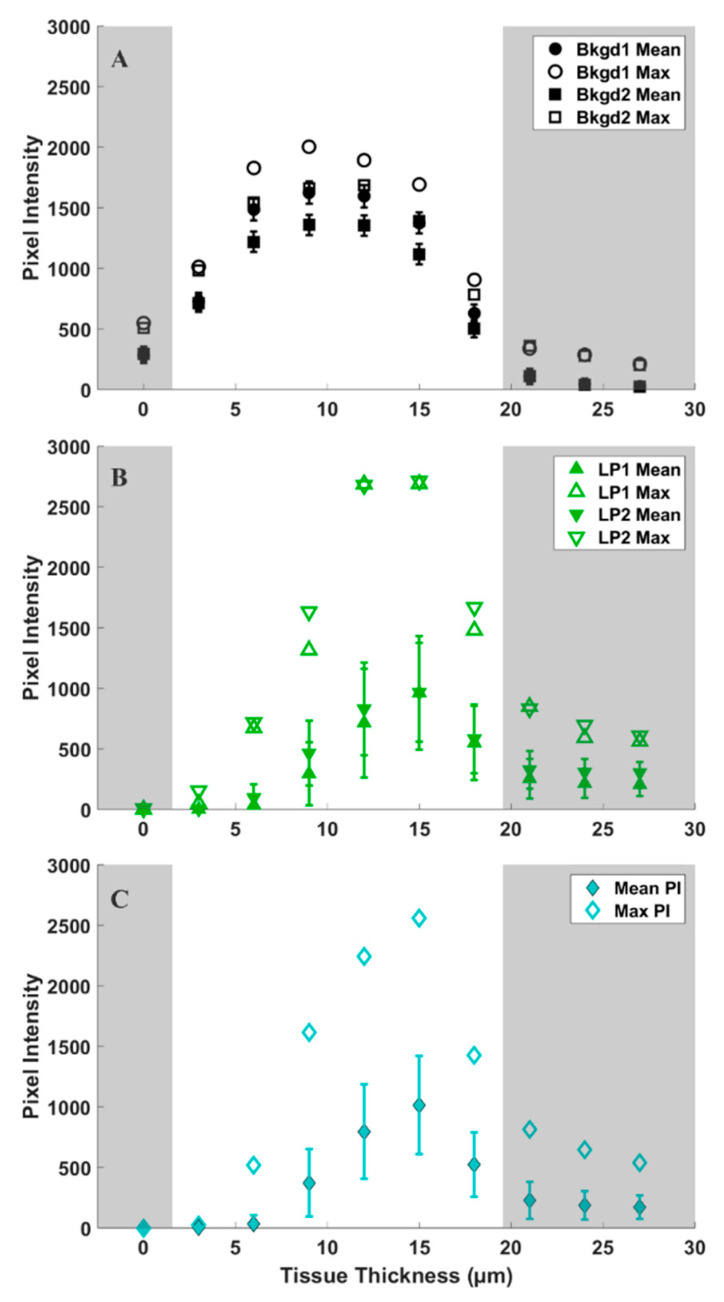
Mean (solid symbols) and maximum (open symbols) pixel intensities of 5400 data points over tissue thickness (shown in Figure 3) for (**A**) background (black), (**B**) leaf primordia (green), and (**C**) meristem (cyan) regions of interest (ROIs) identified in Figure 4A. Error bars represent standard deviations in pixel intensity for each ROI. Shaded regions represent areas of low signal intensity due to technical aspects of CARS imaging (see Supplementary Materials) and are therefore eliminated from our analyses.

To provide a quantitative assessment of the d_6_-DMSO in CARS images shown in Figure 3, we evaluated mean and maximum pixel intensities in specifically chosen regions of interest (ROI). Figure 4A shows a representative CARS image (see Figure 3, 15 μm) where two regions within the leaf primordia, one within the meristem, and two in the background, or regions devoid of tissue, were selected for quantification. Each ROI shown contains a range of pixel intensities that correspond to the d_6_-DMSO concentration within. From these ROIs, we determined the mean and maximum pixel intensities for leaf primordia, meristem, and background regions across all tissue thicknesses from Figure 3 (Figure 4B and Figure 5). We observe the highest mean pixel intensities within tissue regions at a depth of 15 μm while the background regions show consistent high mean intensities over the 6–15 μm depth range (Figure 4B). 

We observe more variation in the d_6_-DMSO signal within tissue regions compared to the fairly consistent signal observed in the background regions. Figure 5 shows the maximum and mean pixel intensities recorded for each ROI over the tissue thicknesses reported in Figure 3. The standard deviations highlight the variation in d_6_-DMSO concentration for each selected ROI. Background regions show relatively small standard deviations in pixel intensity that are consistent across all tissue thicknesses (Figure 5A), while we observe an increasing standard deviation in pixel intensity at central tissue thicknesses for both leaf primordia (Figure 5B) and meristematic (Figure 5C) regions. Maximum pixel intensities appear much farther from the mean values in the plant tissue regions compared to the background regions, but maximum pixel intensities generally still lie outside one standard deviation from the mean for all ROIs. 

**Figure 6:**
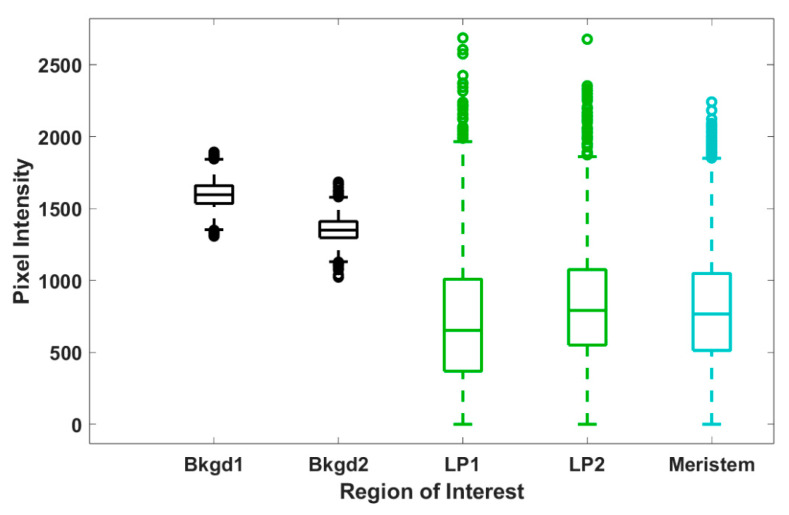
Box and whisker plots for the spread of pixel intensity for each ROI (representative image shown in Figure 4A) at 12 µm tissue thickness (image shown in Figure 3). Each data set contains 5400 data points, corresponding to the 5400 pixel intensities within each ROI; outliers are reported as individual data points. The reported number of outliers for Bkgd1, Bkgd2, LP1, LP2, and meristem regions of interest is 5, 16, 21, 36, and 21, respectively.

To further assess trends in pixel intensity, we performed a statistical analysis across each ROI, representing all pixel intensities, for each ROI at the 12 μm central tissue thickness. Levene’s test for equality of variances shows that all but two ROIs shown have statistically different variances (see Supplementary Materials); however, the spread of the data shown in Figure 6 appears quite uniform across both background and tissue regions. Hence, we calculated both the range and interquartile ranges, reported in Table 1, to confirm consistency in the spread of our data. From these calculations, we make some key observations. For each shoot tip, the full range and interquartile range of background regions are quite similar. Likewise, the range and interquartile range of each tissue region are also similar to each other. We observe in Table 1 that the range and interquartile ranges from the tissue regions are substantially larger than the background regions, further supporting our interpretation from Figure 6. However, the most significant finding is that, except for shoot tip 3 (see Supplementary Materials), the full and interquartile ranges for tissue regions are quite consistent and notably larger than those of the background regions. The lower full and interquartile range values reported for shoot tip 3 (see Supplementary Materials) are likely due to reduced signal from the thickness of the plant tissue, a sample much thicker than all others reported. This suggests the same spread of pixel intensities across tissue and background regions for all shoot tips and, therefore, the same variation in d_6_-DMSO concentration across these respective regions for all shoot tips. Further, the consistency of these data across samples indicates that this analysis may be used as a metric for reproducibility in future work.

## 3. Discussion

In this study, we report the permeation of DMSO into living peppermint shoot tips using both CARS and brightfield microscopy techniques. Together, these microscopies provide non-invasive imaging showing the overall response of peppermint shoot tips to aqueous DMSO as well as evidence of the localization of DMSO within the shoot tips. 

We use brightfield microscopy to investigate the response of peppermint shoot tips to 15% (*w*/*v*) aqueous DMSO. Previously, our group reported the response of individual rice callus cells to aqueous DMSO [34]. Here, we explore the overall response of complex plant tissue, that is, shoot tips comprising much smaller cells (~5 μm diameter), to DMSO exposure. Within the first 5 min of exposure to DMSO, we observe either an initial contraction and re-expansion or just a slight expansion of the entire shoot tip. The initial contraction and expansion responses observed may be the result of increased osmotic pressure on the system. Previous studies have reported the contraction of meristematic regions when exposed to increased osmotic pressure, whereas leaf primordia regions expand under the same stress [36,37]. The subcellular motion observed in some of our samples may indicate plasmolysis and deplasmolysis within individual cells in the shoot tips, likely a result of dehydration that occurs when water exits the cytoplasm [14,15,16]. It has been suggested that the time it takes for plasmolysis and deplasmolysis to complete indicates the time required for DMSO to fully localize within cells [38]; however, CARS microscopy results show the presence of DMSO in the shoot tip well before the shoot tip contraction and expansion responses are complete. The expansion and/or contraction response may be due to the high concentration of solute (DMSO) introduced to the system, while long-term swelling of the shoot tips may result from fluid build-up in the apoplast. Furthermore, DMSO has been reported to disrupt cell wall structure [39], likely reducing the stiffness of the cell walls and contributing to the long-term expansion we observe.

We use CARS microscopy to directly measure DMSO entering plant shoot tips. The CARS data presented here show the distribution of DMSO across and throughout a living shoot tip. We observe that DMSO penetrates deeply into the peppermint shoot tip, with the highest concentrations of DMSO, indicated by the highest CARS signals, coming from the central regions of the shoot tip. In addition, the CARS intensity data show that when it permeates, DMSO is non-uniformly distributed across and throughout the shoot tip, sometimes appearing at a higher intensity than the 15% aqueous DMSO exposed to the sample (Figure 3, Figure 4, Figure 5 and Figure 6, Table 1). The varied CARS intensity suggests that the cryoprotectant may become concentrated in some cells, portions of cells, or between cells. 

Although DMSO permeates into the middle of the shoot tip, it does not appear in the shoot tip periphery (Figure 3). On the timescale of this experiment, we observe that DMSO easily permeates into the central parts of both leaf primordia and meristem regions, which must occur by lateral diffusion through the plant tissue. Because the shoot tip remains stationary during flow, we know that it adheres to the bottom of the flow chamber. This could block the direct perfusion of DMSO into the shoot tip due to its contact with the glass coverslip, blocking the flow of DMSO into the plant tissue. To permeate into the bottom of the shoot tip, DMSO would have to diffuse laterally through the sides of the shoot tip. 

The low CARS signal intensities collected from the top and bottom of the shoot tip also arise from technical issues associated with generating and collecting CARS signals. Because we collect the CARS signal in the forward direction, light must penetrate and traverse the shoot tip to be detected. Differences in refractive index between flow chamber materials (bottom and top glass coverslips, plant material, and/or aqueous solution) impact imaging characteristics, leading to a reduced signal [40,41]. Low or zero CARS signal arising from DMSO in regions without plant tissue, that is, background regions, near the top and bottom of the z-stack indicate that signal reduction occurs from the technical aspects of CARS microscopy rather than from a lack of DMSO in the sample. Thus, we focus our analysis on images in the central portions of each shoot tip measured. A more complete description of these technical issues appears in the Supplementary Materials. 

Differences exist between maximum pixel intensities in background and plant tissue regions, as shown in Figure 3, Figure 4, Figure 5 and Figure 6. The maximum pixel intensities reported for leaf primordia and meristematic regions (from 12–18 μm tissue thicknesses in Figure 5) clearly exceed their standard deviations, but so do the maximum pixel intensities in background regions. However, outliers in tissue regions are skewed in the sample distributions, whereas outliers in background regions are evenly distributed above and below the mean (Figure 6). Furthermore, based on our calibration bar (Figure 3, right), we observe DMSO concentrations as high as ~22% (*w*/*v*) within imaged tissue areas. Taken together, these observations suggest that there may exist regions of concentrated DMSO within the shoot tip. We observe similar trends across all shoot tips studied (see Supplementary Materials).

It is challenging to obtain a quantitative measure of concentration from CARS microscopy signal strength. The quadratic dependence of the CARS signal with concentration as well as contributions from non-resonant background, artifacts, and signal reduction due to either or both sample thickness and refractive index mismatch make it difficult to obtain quantitative data [33,40,42]. Despite these challenges, we estimate DMSO concentrations using a calibration curve generated from standardized samples (Figure S2). From our reported pixel intensities, we estimate DMSO concentrations as high as ~22% (*w*/*v*) within the shoot tip, substantially greater than the 15% (*w*/*v*) aqueous DMSO that flowed over the sample. These results suggest that DMSO is sequestering into localized areas within the shoot tip. This is further supported by the data shown in Figure 5B,C, where we observe much higher maximum pixel intensities in both the meristem and leaf primordia regions compared to those of the background regions (Figure 5A). We also show that outliers in pixel intensity values for tissue regions appear to be skewed above the sample distribution, whereas outliers in pixel intensity values for background regions appear to be evenly distributed above and below (Figure 6). These data indicate that DMSO preferentially resides in certain regions within or between cells in the meristem and leaf primordia. In previous work with rice callus cells, we also observed pooling of DMSO into rice callus cell organelles, attributed to starch granules [34]. The regions of higher intensity we report here may indicate that DMSO pools preferentially in specific organelles, although additional studies are needed.

Researchers and practitioners have used DMSO by itself [43,44] or as a component of PVS2 [8,9,10] to help plants and other cells and tissues withstand the low temperatures associated with cryopreservation, but the actual role of these components in the vitrification process is not well defined. Among other things, previous studies have not identified where cryoprotectant molecules localize in complex plant tissues during the preservation process. Here, we directly demonstrate the location of DMSO within living peppermint shoot tips. Understanding CPA localization may provide further insights into the individual and eventual combined interactions between cryoprotectants and both cell components and intracellular water. Enlisting the combination of a unique vibrational probe, cryoprotectant deuteration, with CARS microscopy, we directly observe pooling of d_6_-DMSO into pockets within plant tissue, potentially in specific cell organelles. We are particularly interested in whether cryoprotectants permeate the cell nucleus. The methods we report here can confirm the specific location of CPAs within individual cells, including in subcellular organelles. Previous studies suggest that pooling or higher concentrations of DMSO could lead to greater water–DMSO interactions [45], stronger interactions with proteins [26,27,28], or even the denaturation of DNA, RNA, and proteins at extremely high concentrations [6,27,46,47], although a recent study suggests high genetic stability for mint cryopreserved with PVS2 [6]. The results we report here support the critical role of DMSO in the preservation process.

## 4. Materials and Methods

### 4.1. Sample Preparation

#### 4.1.1. *Mentha × piperita* Cultures

In vitro *Mentha* × *piperita* L. cv. Todd Mitcham Peppermint (PI 557973) cultures were provided by the USDA National Laboratory for Genetic Resources Preservation (NLGRP; Fort Collins, CO, USA) and propagated on solid medium. Medium was prepared by dissolving 4.43 g L^−1^ Murashige and Skoog basal plant medium with vitamins (MS Basal Medium, M519; PhytoTechnology Laboratories, Lenexa, KS, USA), 15.0 g L^−1^ sucrose (Alfa Aesar, Ward Hill, MA, USA), and 7.00 g L^−1^ agar (BD Diagnostics, Franklin Lakes, NJ, USA) in distilled water, pH 6.1, prior to autoclaving [8,11,48]. Cultures were grown in a growth chamber at 25 °C under LED grow lights with a 16 h day length. After 8–10 weeks, the cultured plants were ready for use.

#### 4.1.2. Cryoprotectant Solutions

Both dimethyl sulfoxide (DMSO) and deuterated DMSO (d_6_-DMSO) solutions were used in these experiments. The 15% (*w*/*v*) DMSO solution was prepared by dissolving DMSO (Fisher Scientific, Fair Lawn, NJ, USA) in distilled water. In addition, 5, 10, 15, and 20% (*w*/*v*) d_6_-DMSO solutions for calibrations were prepared by dissolving d_6_-DMSO (MilliporeSigma, St. Louis, MO, USA) in distilled water. 

#### 4.1.3. Shoot Tip Excision and Sample Preparation

Single, 2.5 cm nodal sections were placed onto solid MS basal medium and grown at 25 °C on a 16 h light schedule for 3 d prior to shoot tip excision. Shoot tips (0.02–0.30 mm^2^) were excised from the 1–2 mm lateral buds formed on single nodal sections using a dissecting microscope (Leica model MS5; Leica Biosystems, Nussloch, Germany) [11].

### 4.2. Microscopy and Spectroscopy Methods

#### 4.2.1. Brightfield Microscopy with Flow

Brightfield microscopy experiments were conducted using an inverted Olympus IX73 fluorescence microscope (Olympus Corporation, Tokyo, Japan). Flow experiments utilized rudimentary perfusion chambers as described in Samuels et al. [34]. However, 1% poly-L-lysine was not necessary due to the ‘sticky’ nature of the plant shoot tip tissue. Shoot tips were excised and transferred to perfusion chambers with a droplet of liquid MS basal medium (MS basal medium + 15 g L^−1^ sucrose) and a coverslip placed on top to seal the chamber. Flow was induced by placing a droplet (~0.5 mL) of 15% (*w*/*v*) DMSO on a single edge of the coverslip and a KimTech wipe on the other edge. Flow experiments were recorded in real time with a series of images taken over 30 min at 5 s intervals for a total of 361 frames. All images were collected through the microscope’s 40×, 0.60 NA air immersion objective. 

#### 4.2.2. Steady-State Raman and IR Spectroscopy

Spontaneous Raman spectra of DMSO and d_6_-DMSO were collected with 532 nm excitation from 1000–4000 cm^−1^ (Horiba TeraHertz Raman System, Horiba, Kyoto, Japan) [34]. Spectra were normalized for comparison to each other. IR absorption spectra were collected using a Hyperion 3000 FT-IR microscope (Bruker, Berlin, Germany) equipped with a germanium-tipped ATR 20× microscope objective. Samples were placed on 25 mm calcium fluoride plates (Crystrand Ltd., Dorset, United Kingdom) and scanned 64 times with 2 cm^−1^ resolution to generate spectra.

#### 4.2.3. CARS Microscopy Studies

Shoot tip samples for CARS experiments were excised and transferred to rudimentary perfusion chambers 12–24 h before conducting CARS experiments. (Because CARS microscopy experiments occur a 70 min drive from Colorado State University, samples are prepared one day in advance. Staining with Evans blue dye indicated that excised shoot tips remained viable in perfusion chambers for up to 24 h). Two drops of liquid MS basal medium were placed on each side of the coverslip, placed into a 50 mm Petri dish, and sealed with two layers of plastic wrap to minimize contamination and media evaporation. Prior to initial CARS imaging, a separate set of experiments was conducted to determine sample viability within these prepared perfusion chambers (see Supplementary Materials).

CARS microscopy studies were performed at the Advanced Light Microscopy Core (University of Colorado, Denver). The microscope has been previously described [34]. Briefly, CARS images were acquired using an inverted Olympus microscope with laser excitation from an APE picoEMERALD fiber laser that generates pulses at an 80 MHz repetition rate centered at 1030 nm (Stokes) and an optical parametric oscillator that provides the pump beam tuned to 845.5 nm to detect the C-D stretch of d_6_-DMSO at 2125 cm^−1^. The two beams are overlapped spatially and temporally before entering the microscope and impinging on the sample using a 20×, 0.75 NA air-immersion objective. The CARS signal is collected in the forward direction through a 705–755 nm bandpass filter prior to detection on a photomultiplier tube. An additional channel in the epi-direction monitors autofluorescence from the sample; a bandpass filter in the epi-direction enables light detection in the 500–570 nm range. Images and z-stacks were recorded using FV10-ASW 4.2 acquisition software (Olympus Corporation, Tokyo, Japan). To ensure CARS signals arise only from the target CPA, that is, d_6_-DMSO, we eliminated various interfering signals and describe the process in the Supplementary Materials. 

Shoot tips were placed in perfusion chambers and directly mounted on the CARS microscope sample stage. Liquid MS basal medium was flowed over each sample to ensure that each shoot tip remained stationary during and after flow. A brightfield image was collected prior to each CARS experiment. Following brightfield microscopy, 15% (*w*/*v*) d_6_-DMSO was flowed through the perfusion chamber on the microscope stage. A series of images taken at multiple focal planes along the z-axis of the sample, or z-stacks, were collected in all three of the microscope’s collection channels before and after 10 min of 15% (*w*/*v*) d_6_-DMSO exposure. In these experiments, z-stack images were collected from top to bottom of the sample in 3 µm steps (see Supplementary Materials for more detail). As the size of each shoot tip varies, the total number of images, or “slices”, in each z-stack varies. Background z-stacks were also collected for all three channels by detuning the laser as described above. In some cases, a brightfield image was also collected after 15% (*w*/*v*) d_6_-DMSO flow.

### 4.3. Image and Data Analysis

All images were analyzed using ImageJ (ImageJ ver. 1.53a; Wayne Rasband, National Institutes of Health, Bethesda, MD, USA). 

#### 4.3.1. CARS z-Stack Processing

The top and bottom of each shoot tip, defined in Figure S9, were determined from two-photon autofluorescence detected in the epi-channel at 500–575 nm. Frames collected above and below the shoot tip in both epi-channels and the forward CARS channel were not used in our analysis. 

To ensure the signals reported reflect only the CARS signals arising from d_6_-DMSO, we measured the background for this channel in three different ways. First, we measure any signal arising from the illumination of the sample prior to exposure with d_6_-DMSO and with Stokes and pump laser beams detuned, *I_detuned_*. Second, we measure the same sample but with Stokes and pump laser beams impinging at the same time, which results in a non-resonant CARS signal, *I_NR-CARS_*. Third, we measure the sample after d_6_-DMSO exposure with Stokes and pump laser beams detuned, *I_detuned-DMSO_*. This third configuration can yield two-photon fluorescence but not non-resonant CARS. We have observed that exposing shoot tips to 15% (*w*/*v*) d_6_-DMSO leads to increased *I_detuned-DMSO_* compared to *I_detuned_*. Both *I_detuned-DMSO_* and *I_NR-CARS_* should include the intensity associated with *I_detuned_*. Thus, to extract the true CARS signal, in the absence of all background contributions, we process each pixel intensity as:

(1) $$I_{signal}=\left(I_{CARS}-I_{NR-CARS}\right)-\left(I_{detuned-DMSO}-I_{detuned}\right),$$

where *I_CARS_* is the signal collected from samples exposed to d_6_-DMSO and measured with Stokes and pump laser pulses overlapped in time. Subtracting *I_detuned_* from *I_detuned-DMSO_* ensures that we have only removed the contribution from increased autofluorescence and not the contribution from *I_detuned_* twice. 

The brightness and contrast of the maximum intensity projection image were adjusted, and the pixel intensity range was noted. This pixel intensity range was used to apply the same brightness and contrast adjustment to all individual images of the z-stack simultaneously before individual images were isolated.

#### 4.3.2. Region of Interest Analysis

To compare the signal intensity in the images collected, we identified regions of interest (ROIs) and computed the mean intensity and standard deviation. Boxes that define the ROIs each enclose exactly 5400 pixels and represent regions of the background, leaf primordia, and meristem. Area, standard deviation, and pixel intensity values for each ROI were generated by ImageJ for all slices of the final CARS z-stack. 

#### 4.3.3. Statistical Analysis

We conducted Levene’s statistical test to determine the statistical significance between sample variances for each ROI within a central tissue thickness of multiple shoot tips. More information about Levene’s test and its results can be found in the Supplementary Materials.

All the data presented in Table 1 and Table S1 were generated using custom code written in MATLAB (version R2022b). Levene’s test was conducted using a code developed in 2003 by Trujillo-Ortiz and Hernandez-Walls [49].

## Figures and Tables

**Table 1:** Range and interquartile range (IQR) for each region of interest (ROI) of each shoot tip presented *.

Shoot Tip	Region	Range (Pixel Intensity)	IQR (Pixel Intensity)
ST1	Bkgd1	584	124
ST2		679	132
ST3		708	127
ST1	Bkgd2	661	113
ST2		651	125
ST3		696	119
ST1	LP1	2686	639
ST2		2646	596
ST3		1525	245
ST1	LP2	2677	524
ST2		2625	455
ST3		1244	201
ST1	Meristem	2241	535
ST2		2453	450
ST3		1849	375

* ST = shoot tip; ST1 data appear in main text; ST2 and ST3 data appear in Supplementary Materials.

## Data Availability

All data are available in the main text or the Supplementary Materials.

## Supplementary Materials

The following supporting information can be downloaded at: https://www.mdpi.com/article/10.3390/plants12122247/s1, Figure S1: Response of additional peppermint shoot tips to aqueous DMSO using brightfield microscopy; Figure S2: CARS calibration curve; Figure S3: CARS montage for a second shoot tip exposed to aqueous DMSO; Figure S4: CARS montage for a third shoot tip exposed to aqueous DMSO; Figure S5: Representative CARS images and mean pixel intensity vs. tissue thickness for the two additional shoot tips shown in Figures S3 and S4; Figure S6: Mean and maximum pixel intensities vs. tissue thickness for each ROI for the two additional shoot tips shown in Figures S3 and S4; Figure S7: Box and whisker plots for each ROI at a central depth for the two additional shoot tips shown in Figures S3 and S4; Figure S8: Evans blue tissue staining; Figure S9: Cartoon defining orientation of the shoot tip relative to CARS microscope; Table S1: Results of Levene’s test for sample variances.
