# Supplementary material for: Tracking Permeation of Dimethyl Sulfoxide (DMSO) in Mentha × piperita Shoot Tips Using Coherent Raman Microscopy
Source: Plants (Basel). 2023 Jun 8;12(12):2247. doi: 10.3390/plants12122247 (PMC10304857; doi:10.3390/plants12122247)
Supplement: Supplementary file 1 [file plants-12-02247-s001.zip › plants-2313881-supplementary.pdf]

plants

MDPI

Supplementary Materials

1. Brightfield Imaging

We conducted six brightfield flow experiments to determine the reaction of whole peppermint shoot tips to aqueous DMSO exposure. Figure S1 shows the expansion of two additional shoot tips after 20–25 min of exposure to 15% (*w*/*v*) aqueous DMSO. All shoot tips we measured demonstrated some degree of expansion, although some expanded more than others, supporting the results presented in Figure 1.

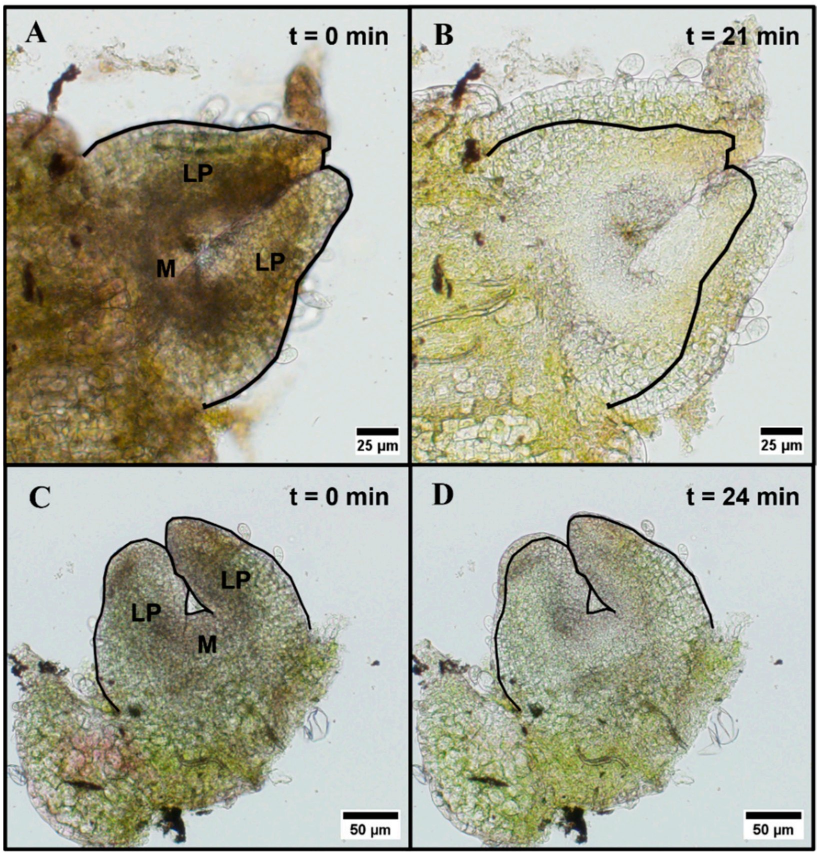

Figure S1. Response of peppermint shoot tips to 15% (w/v) DMSO: (**A**,**C**) two different shoot tips before exposure to DMSO solution (**B**,**D**) The same shoot tips 21 min and 24 min after exposure to DMSO solution, respectively. LP = leaf primordia region, M = meristem region. Black lines indicate the edge of each shoot tip prior to exposure to DMSO, and lines are copied to panels showing shoot tips after exposure. Images were collected using a 40×, 0.60 NA air-immersion objective. Image size: 1600 × 1200 pixels. Pixel size: 0.11 µm/pixel.

2. CARS Calibration

To estimate aqueous d_6-DMSO concentrations for the CARS images presented in this study, we developed a calibration curve to correlate pixel intensity to approximate concentration (**Figure S2**). The calibration curve was obtained by collecting images of liquid MS basal medium plus 15 g L^{-1} sucrose and of 5, 10, 15, and 20% (w/v) d_6-DMSO solutions in the forward-CARS channel for a total of four calibrations/solution. To match the focal distance of the CARS microscope (~1 mm), calibration chambers were prepared by sandwiching a droplet (~0.5 mL) of each solution between a 25 mm ′ 50 mm coverslip and a 22.5 mm ′ 22.5 mm coverslip with a 0.25 mm sticky spacer (SunJin Lab Co., Hsinchu City, Taiwan, R.O.C.). Each calibration chamber was measured at the maximum of the d_6-DMSO signal, as described in the text. The results were then fitted to a second-order polynomial, yielding the relationship $Mean\ pixel\ intensity = 3.19[DMSO]^2 + 51.3[DMSO] +$

5.43×10^{-23} with $R^2 = 0.9939$. We use this equation to approximate the d6-DMSO concentration in various parts of the shoot tips. In some places, the d6-DMSO concentration appears to exceed the 15% (w/v) concentration of the solution used to expose the shoot tips.

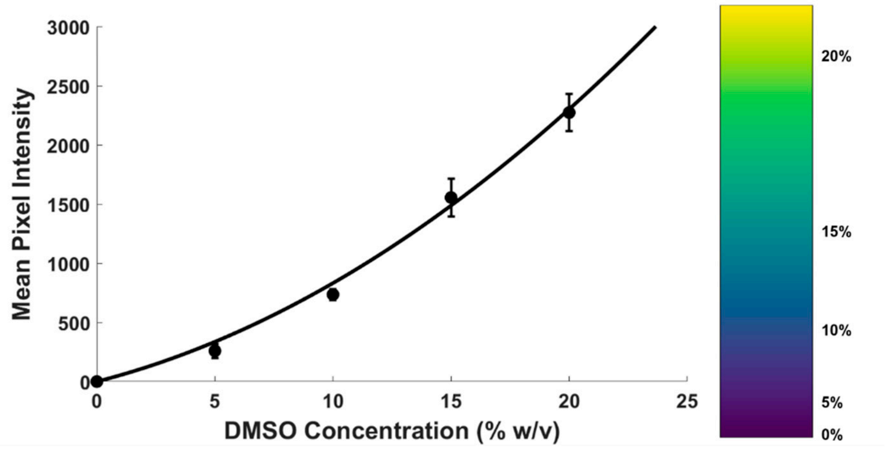

Figure S2. Left: calibration curve of mean pixel intensity vs. d6-DMSO concentration for 0, 5, 10, 15, and 20% (*w*/*v*) aqueous d6-DMSO (points) and quadratic fit (line). Error bars represent the standard deviation from 2–4 measurements. Right: calibration bar shows estimated d6-DMSO concentrations from pixel intensity values correlated to viridis color scheme used in images shown in Figures 2, 3, S3, and S4.

3. CARS Imaging

To confirm and support the results reported in **Figures 3–5,** we present CARS microscopy data for two additional shoot tips. **Figures S3–S4** represent CARS z-stack montages for those two additional shoot tips exposed to aqueous d6-DMSO. The figures show that d6-DMSO penetrates into the interior of each shoot tip, similar to the shoot tip shown in the main text. Differences in shoot tip thickness alter the specific behavior.

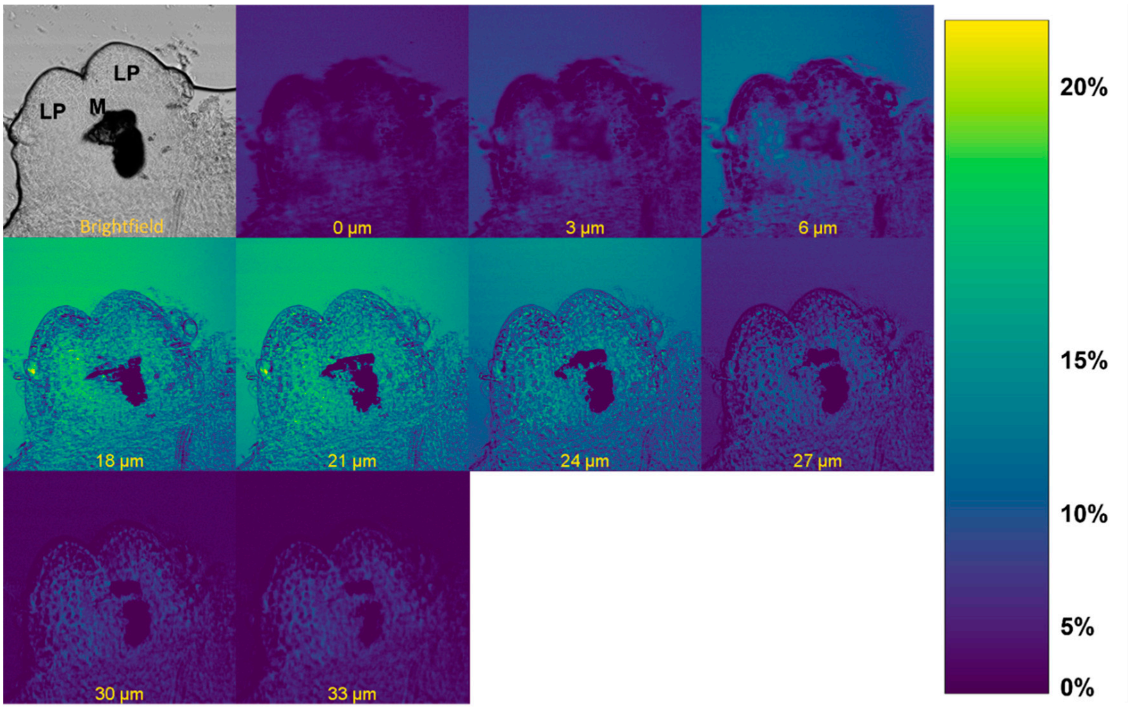

Figure S3. CARS montage for a z-stack of a peppermint shoot tip following >10 min exposure to 15% (*w/v*) d_6-DMSO in MS media. **Top left:** brightfield image of the shoot tip identifying leaf primordia (LP) and meristem (M) regions. **All others:** CARS images taken at depths of 0, 3, 6, 18, 21, 24, 27, 30, and 33 µm. The second panel from the left in the top row represents an image taken from the top of the shoot tip; subsequent images are presented in order from top to bottom of the shoot tip. Calibration bar from Figure S2 (right) shows approximate d_6-DMSO concentrations. Images were collected using a 20×, 0.75 NA air immersion objective. Vibrational frequency: 2125 cm^{-1}. Image size: 800 × 800 pixels. Pixel size: 0.12 µm/pixel. Scale bar = 10 µm.

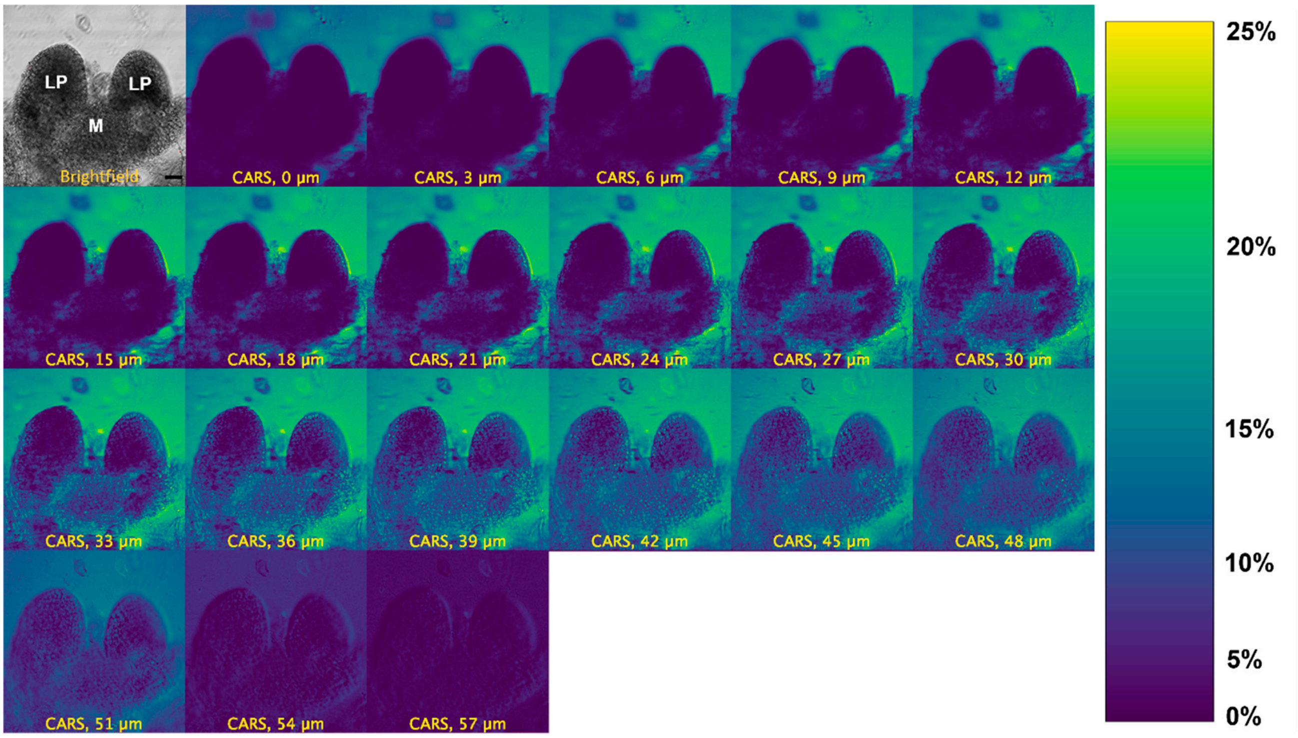

Figure S4. CARS montage for a z-stack of a peppermint shoot tip following >10 min exposure to 15% (w/v) d_6-DMSO. **Top left:** brightfield image of the shoot tip identifying leaf primordia (LP) and meristem (M) regions. **All others:** CARS images taken at depths of 0, 3, 6, 9, 12, 15, 18, 21, 24, 27, 30, 33, 36, 39, 42, 45, 48, 51, 54, and 57 µm. The second panel from the left in the top row represents an image taken from the top of the shoot tip; subsequent images are presented in order from top to bottom of the shoot tip. (The final three spaces following the image shown at 51 µm are placeholders and contain no data). Calibration bar from Figure S2 (right) shows approximate d_6-DMSO concentrations. Images were collected using a 20×, 0.75 NA air immersion objective. Vibrational frequency: 2125 cm^{-1}. Image size: 800 × 800 pixels. Pixel size: 0.12 µm/pixel. Scale bar = 10 µm. We analyze the CARS images of the two additional shoot tips, shown in **Figures S3** and **S4**, in the same way as presented in the main text. Figures S5B and D and Figures S6B and D report mean and maximum pixel intensities for indicated regions (Figure S5A and C; Figure S6A and C) of each shoot tip corresponding to background (media, aqueous d_6-DMSO), leaf primordia, and the meristem. Images were collected using a 20×, 0.75 NA air immersion objective. Vibrational frequency: 2125 cm^{-1}. Image size: 800 × 800 pixels. Pixel size: 0.12 µm/pixel. Scale bar = 10 µm.

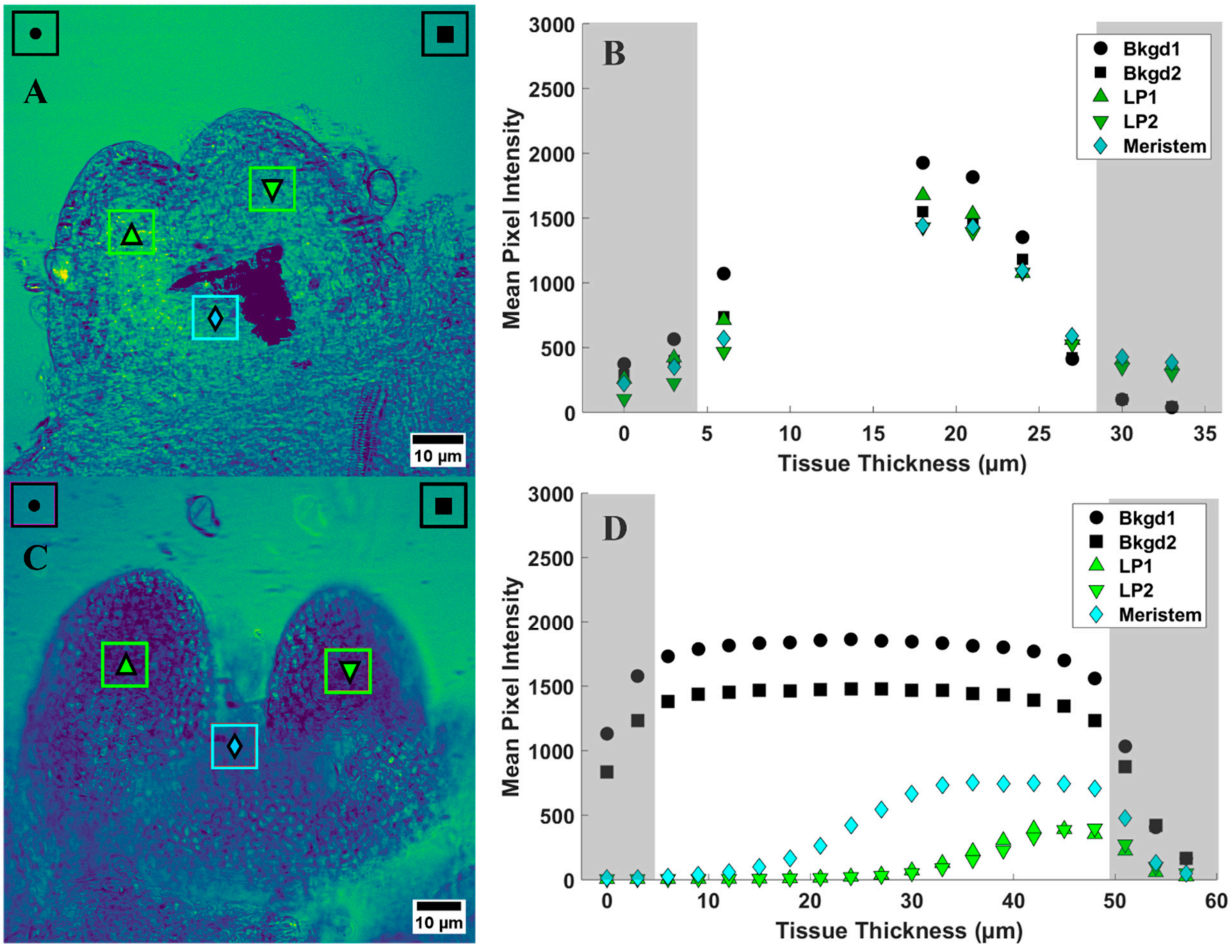

Figure S5. (**A)** Representative image at 21 µm from z-stack shown in **Figure S3** with background (black), leaf primordia (green), and meristem (cyan) regions of interest indicated; (**B)** mean pixel intensity over tissue thickness for each ROI shown in **A**; **(C**) representative image at 39 µm from z-stack shown in **Figure S4** with background (black), leaf primordia (green), and meristem (cyan) regions of interest indicated; (**D)** mean pixel intensity over tissue thickness for each ROI shown in C. Shoot tips were exposed to 15% (w/v) d_6-DMSO. Shaded regions represent areas of low signal intensity due to technical aspects of CARS imaging and are thereby eliminated from our analyses. Images were collected using a 20×, 0.75 NA air immersion objective. Vibrational frequency: 2125 cm^{-1}. Image size: 800 × 800 pixels. Pixel size: 0.12 µm/pixel. Scale bar = 10 µm.

Figure S5 shows representative images of each shoot tip shown in Figures S3 and S4, indicating each region of interest (ROI) and the resulting mean pixel intensities for each ROI over depth of each shoot tip. The highest intensities were observed in central regions of each shoot tip: 12–20 µm (Figure S5, B) and 30–50 µm (Figure S5, D). In each case, the mean background intensity exceeds the mean intensity for the shoot tip tissue region. The missing data points in Figures S5B and S6A–C are attributed to a shutter closure during data acquisition.

In addition to mean pixel intensity, we demonstrate that the leaf primordia and meristem regions have a greater variation in pixel intensities in these central regions, as demonstrated by the standard deviations shown, with their maximum pixel intensities drastically increasing, unlike the background regions, where intensity variation and maximum pixel intensities remain relatively consistent (Figure S6).

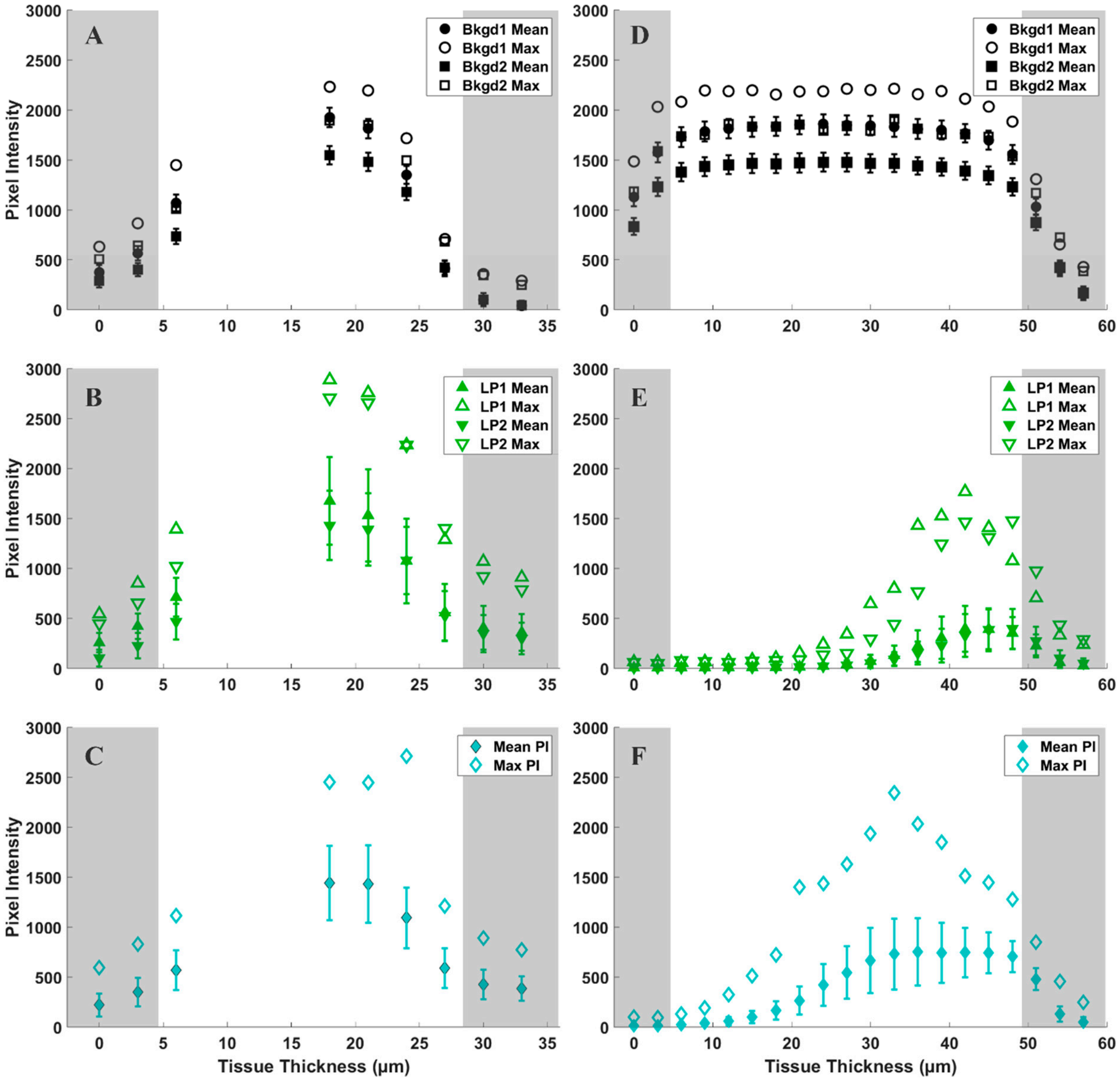

Figure S6. Mean and maximum pixel intensities over tissue thickness. Plots A–C report pixel intensities in the shoot tip shown in **Figure S3**. Plots D–F report pixel intensities in the shoot tip shown in **Figure S4**. Panels A and D report pixel intensities in background ROIs; panels B and E report pixel intensities in leaf primordia ROIs; panels C and F report pixel intensities in meristem ROIs for each shoot tip shown in **Figures S3 and S4**. Shaded regions represent areas of low signal intensity due to technical aspects of CARS imaging and are thereby eliminated from our analyses.

4. CARS signal intensity

The z-stacks that we present in Figures 3, 4, S3, and S4 display a strong variation in overall signal intensity.

The laser excitation leading to the d_6-DMSO CARS signal is focused into the sample with a 20×, 0.75NA air objective, which yields a sectioning capability in the z-direction (top to bottom of the sample) of approximately one-third of the typical sample thickness (10–15 μm). Thus, the CARS signals we collect represent an average of several layers in the sample. Images acquired at the top and bottom of the sample average over layers outside the plant tissue, including the glass coverslips that contribute no CARS signal.

The primary loss of signal occurs due to the deteriorating focus of the laser beams responsible for the CARS signal. The used air objective is optimized for focusing through

air and a #1.5 coverslip, which yields the best focus right at the glass coverslip/sample interface. When imaging deeper into the sample, the laser light on the way to the focus travels through parts of the sample and/or DMSO, which have a refractive index that is significantly higher than that of air. This results in spherical aberrations in the laser focus that spread out the focus. The spreading of the focus is most pronounced in the z-direction and leads to a lower photon density in the laser focus, which then leads to a smaller CARS signal. This effect is cumulative. Focusing deeper into the sample yields a stronger deterioration of the focus due to spherical aberrations, as will a higher refractive index of the traversed material.

Thus, the laser focus gets worse as the light penetrates deeper into the sample, causing a reduction in the CARS signal and z-resolution. The tissue region has a lower refractive index than the surrounding solution, so we observe a larger decrease of the CARS signal from the background regions than the tissue region (with a lower refractive index) as we approach the top of the sample, which leads to background regions of the images that have lower intensity than the tissue regions near the bottom of the sample.

The strongest CARS signal of the aqueous d_6-DMSO solution coincides with the center layers of the plant tissue. Thus, we restrict our analysis to the center layers of the plant tissue.

5. Statistical Analysis

For further data analysis, we constructed a series of box and whisker plots for each ROI at a central tissue thickness for each shoot tip (Figure S7). For both shoot tips ST2 and ST3, we observe notably larger variance within tissue regions when compared to the background regions, supporting the results presented in the main text.

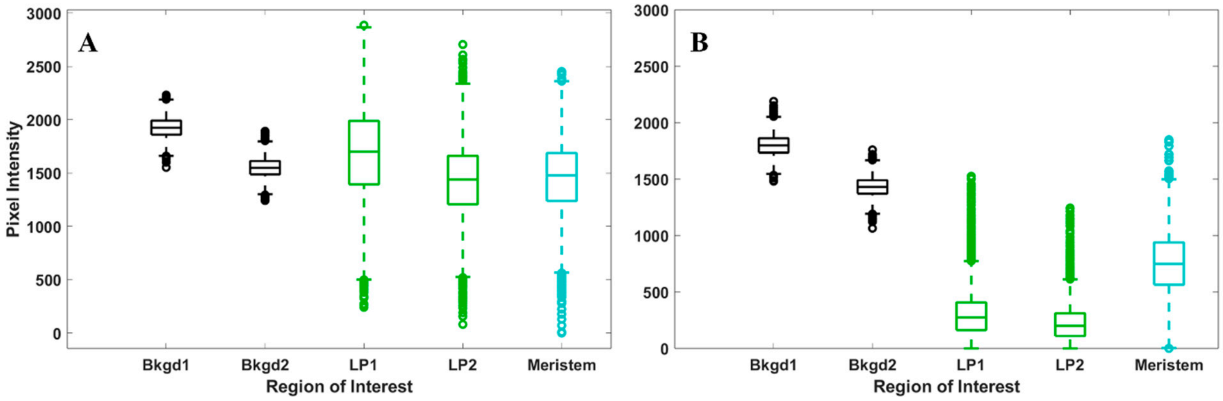

Figure S7. Box and whisker plots for each ROI for (**A**) 18 µm tissue thickness of the shoot tip shown in **Figure S5A**, and (**B**) 39 µm tissue thickness of the shoot tip shown in **Figure S5B**. Each data set contains 5400 data points, with outliers appearing as separate data points. The number of outliers reported for the Bkgd1, Bkgd2, LP1, LP2, and meristem regions of each shoot tip is (**A**) 6, 11, 7, 35, and 73; and (**B**) 11, 13, 145, 121, and 9, respectively. .

To test for statistical significance between the variances of each ROI, we conducted Levene's test for equality of variances. Levene's test is a nonparametric test, meaning the test's results are less sensitive to data departures from normalcy. This was an important factor in our selection because, although the pixel intensity distributions for each ROI in the first shoot tip appear to fit a normal distribution (Figure S7A), the pixel intensity distributions for the tissue ROI in the second shoot tip appear non-normal (Figure S7B).

The results of Levene's test for the shoot tip discussed in the main text, ST1, are shown in Table S1. All p-values less than 0.05 are statistically significant, allowing us to reject the null hypothesis that sample variations are equal. This analysis shows that all but two of the regions of interest tested have statistically different variances; only two regions, the LP2 and meristem, have statistically similar variances. Levene's test results for ST2

and ST3 (not shown) yield statistically different variations with p = 0.0000 between all ROIs.

Table S1. Results of Levene's test for sample variances for ST1, the shoot tip presented in **Figure 3** of the main text. df1 represents the degrees of freedom for the number of data sets. df2 represents the number of degrees of freedom for total data points. Each sample set consists of 5400 data points and corresponds to the data presented in Figure 6.

Group A	Group B	df1	df2	F-value*	p-value
Bkgd1	Bkgd2	1	10800	38.40	0.0000
Bkgd1	LP1	1	10800	6467	0.0000
Bkgd1	LP2	1	10800	5270	0.0000
Bkgd1	Meristem	1	10800	5419	0.0000
Bkgd2	LP1	1	10800	6797	0.0000
Bkgd2	LP2	1	10800	5609	0.0000
Bkgd2	Meristem	1	10800	5758	0.0000
LP1	LP2	1	10800	152.1	0.0000
LP1	Meristem	1	10800	120.6	0.0000
LP2	Meristem	1	10800	1.875	0.1709

* The F-test is the non-parametric equivalent of a t-test, comparing variances rather than the mean values of the sample.

6. Evans Blue Staining

As excised shoot tips were enclosed in flow chambers containing liquid MS media for up to 24 h prior to CARS experiments, it was essential to determine that these shoot tips remained viable. To test this, shoot tips were excised and transferred into flow chambers, sealed with parafilm, and left in the dark for 24 h. After 24 h, the shoot tips were stained with a 0.5% (*w*/*v*) Evans blue solution to determine viability. The 0.5% (*w*/*v*) Evans blue solution was prepared by dissolving 0.05 g of Evans blue (Chem-Impex International, Wood Dale, IL, USA) in 10 mL of distilled water. Shoot tips were incubated in a drop of Evans blue solution for 1 min at room temperature prior to rinsing with liquid media. Stained shoot tips were then imaged using brightfield microscopy.

Evans blue is a dye that perfuses into dead cells/tissues turning them dark blue. Figure S8 shows two shoot tips before (Figure S8A and C) and after (Figure S8B and D) Evans blue staining 24 h after excision. After staining, the exterior regions of the shoot tips appear dark blue, indicating non-viable tissue. This is likely due to tissue damage from excision. However, the leaf primordia and meristem regions, which are key to our studies, still appear clear green, indicating that these cells are still viable in the flow chamber 24 h after excision.

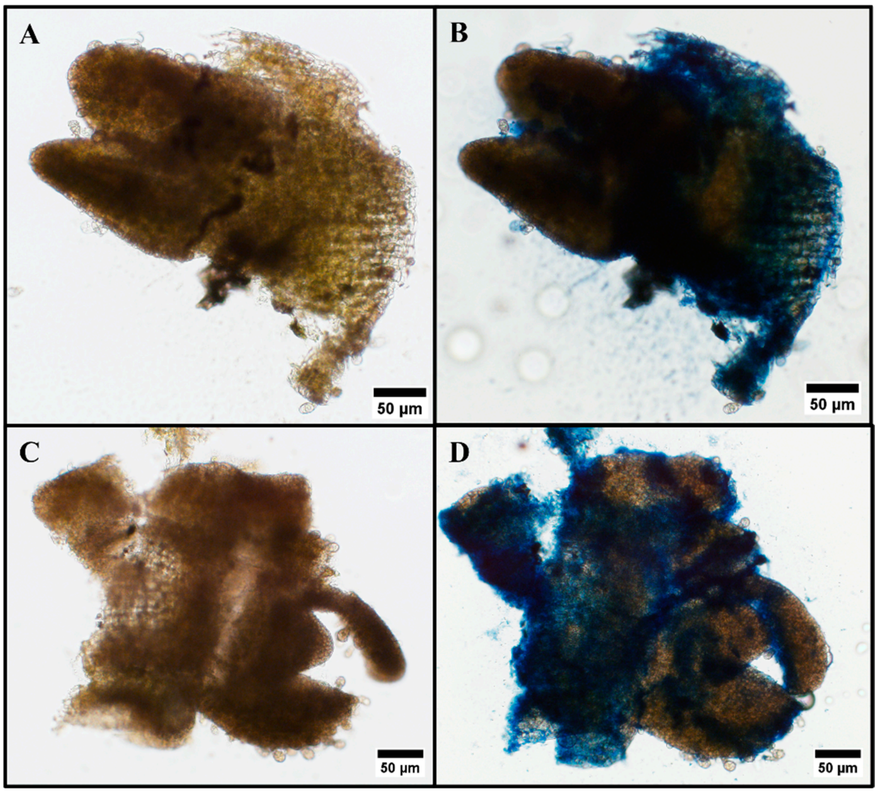

Figure S8. Brightfield microscopy images of peppermint shoot tips (**A**, **C**) before and (**B**, **D**) after Evans blue tissue staining, respectively. All images were taken 24 h after excision.

7. Sample Orientation for CARS Microscopy

We refer to the top and bottom of each shoot tip throughout this work, which we define relative to the microscope orientation. Figure S9 provides a cartoon identifying this orientation, where the top of the shoot tip is defined as being closest to the detection and the bottom corresponds to where the pump and Stokes laser beams first enter the sample. We refer to each image collected in order from the top of the shoot tip to the bottom of the shoot tip, corresponding to increasing tissue thickness. However, laser light entered the sample through the bottom, and the resulting signal was collected over the top of the shoot tip sample, meaning that laser light propagates further through the plant tissue before generating a CARS signal, and the CARS signal generated near the bottom of the sample must propagate through the rest of the sample to reach the top and be detected.

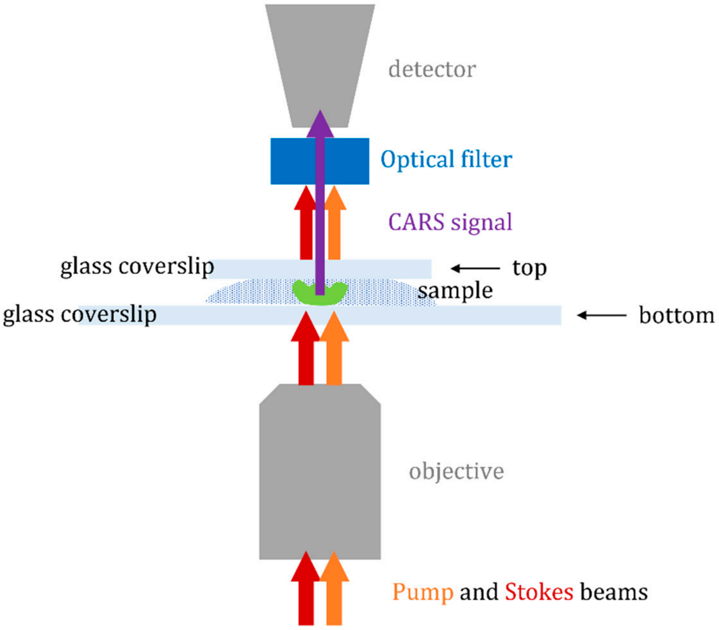

Figure S9. Schematic demonstrating laser light propagation through shoot tips and resulting CARS signal detection. Shoot tip orientation (top and bottom) shows that tissue thickness increases from top to bottom of the shoot tip, where light must travel furthest to reach the top of the sample.
